# Study of Nanohydroxyapatite Coatings Prepared by the Electrophoretic Deposition Method at Various Voltage and Time Parameters

**DOI:** 10.3390/ma17102242

**Published:** 2024-05-10

**Authors:** Klaudia Malisz, Beata Świeczko-Żurek, Jean-Marc Olive, Grzegorz Gajowiec, Gilles Pecastaings, Aleksandra Laska, Alina Sionkowska

**Affiliations:** 1 Department of Biomaterials Technology, Faculty of Mechanical Engineering and Ship Technology, Gdansk University of Technology, Gabriela Narutowicza 11/12, 80-229 Gdansk, Poland; beata.swieczko-zurek@pg.edu.pl; 2 CNRS—Centre National de la Recherche Scientifique, Institute de Mecanique et d’Imgenierie, Universite de Bordeaux, 33405 Talence, France; jean-marc.olive@u-bordeaux.fr; 3 Department of Materials Science and Technology, Faculty of Mechanical Engineering and Ship Technology, Gdansk University of Technology, Gabriela Narutowicza 11/12, 80-229 Gdansk, Poland; grzgajow@pg.edu.pl (G.G.); aleksandra.laska@pg.edu.pl (A.L.); 4 Centre de Recherche Paul Pascal, CNRS Universite de Bordeaux, UMR 5031, 33600 Pessac, France; gilles.pecastaings@crpp.cnrs.fr; 5 Department of Biomaterials and Cosmetic Chemistry, Faculty of Chemistry, Nicolaus Copernicus University in Torun, 87-100 Torun, Poland

**Keywords:** hydroxyapatite, titanium alloy, electrophoretic deposition, wettability, adhesion

## Abstract

The aim of the work is to compare the properties of nanohydroxyapatite coatings obtained using the electrophoretic deposition method (EDP) at 10 V, 20 V, and 30 V, and with deposit times of 2 and 5 min. The primary sedimentation was used to minimize the risk of the formation of particle agglomerates on the sample surface. Evaluation of the coating was performed by using a Scanning Electron Microscope (SEM), Energy-Dispersive Spectroscopy (EDS), Atomic Force Microscopy (AFM), optical profilometer, drop shape analyzer, and a nanoscratch tester. All of the coatings are homogeneous without any agglomerates. When low voltage (10 V) was used, the coatings were uniform and continuous regardless of the deposition time. The increase in voltage resulted in the formation of cracks in the coatings. The wettability test shows the hydrophilic behavior of the coatings and the mean contact angle values are in the range of 20–37°. The coatings showed excellent adhesion to the substrate. The application of a maximum force of 400 mN did not cause delamination in most coatings. It is concluded that the optimal coating for orthopedic implants (such as hip joint implants, knee joint implants or facial elements) is obtained at 10 V and 5 min because of its homogeneity, and a contact angle that promotes osseointegration and great adhesion to the substrate.

## 1. Introduction

One of the most advanced metallic biomaterials used for long-term orthopedic and dental implants is titanium and its alloys [1,2]. The mechanical properties of the Ti-13Zr-13Nb alloy are adequate compared to technical titanium, Ti-6Al-4V and Ti-6Al-7Nb. Additionally, the low elastic modulus of the Ti-13Zr-13Nb alloy (about 80 GPa [3,4]) is closer to that of a bone modulus (18–19 GPa [3]), which decreases the possibility of the screening effect and disappearance of tissue around the implant [1]. In the case of vascular stents, the most commonly used metal alloys for stent platforms are 316 L stainless steel, cobalt-chromium, tantalum, and titanium alloys [5,6,7]. Despite several advantages such as good flexibility, radial strength, and better radiopacity, cobalt-based alloys could indicate cytotoxic effects. In comparison, commercially pure titanium possesses excellent corrosion resistance and biocompatibility properties [5]. Nitinol (NiTi alloy) has gained recognition, but scientists are still developing new materials for applications in interventional cardiology.

Hydroxyapatite (HAp) is a functional, bioresorbable, bioactive, and biocompatible bioceramic. It is one of the main components of natural bone and can increase the concentration of local Ca^2+^ [8,9,10]. Hydroxyapatite is widely used in dentistry due to the possibility of tooth remineralization, reduction in tooth sensitivity, oral biofilm control, and tooth whitening [11]. In addition, it plays an important role in orthopedics because of its properties, including high bone conductivity, biocompatibility, non-immunogenicity, and bioactivity. It has also been used in bone repair [12]. Additionally, HAp can enhance the osseointegration process by promoting rigid anchorage between the implant and the surrounding tissue without the growth of fibrous tissue. A long-lasting bone anchoring is maintained by effective osseointegration, completely restoring functional ability [13]. Additionally, the HAp coating can improve the corrosion resistance of the implant in the human body, which can reduce the metallic ions release [14]. To increase defense against infections, hydroxyapatite coatings can be doped with Ag and Sr. This modification promotes osteoblast proliferation and especially causes increased levels of alkaline phosphatase biomarkers of osteogenesis [15,16]. Additionally, the addition of strontium in the hydroxyapatite coating improves its adhesion to the substrate [17]. Nanoparticles can also be used to improve mechanical properties. Mehrvarz A. et al. [18] deposited HAp coating and nanocomposite coating (HAp with ZnO nanoparticles), using the electrochemical deposition method, on the NiTi alloy using the pulse mode. They assessed its mechanical hardness and tribological performance. The composite was characterized by greater hardness, exhibited more plasticity and better adhesion of the coating to the substrate [18].

Dudek K. et al. [19] proposed a method for the functionalization of a clamp made of NiTi shape memory alloy. They functionalized NiTi in two stages: passivation and hydroxyapatite coating formation. In addition to assessing the morphology, topography, structure, and functionalized deformation ability of the implant, they also focused on the biological response of the hydroxyapatite coating. By applying a voltage of 60 V for 30 s, they obtained a thin (approx. 1.5 μm), uniform coating covering the entire surface of the implant. An important feature of the coating is its ability to follow shape changes caused by shape memory effects. The obtained layer showed resistance to cracking due to deformations related to the induction of the shape memory effect in the implant. However, cells growing on the layer were characterized by high activity of mitochondrial enzymes and excellent adhesion to the substrate [19]. HAp has also been successfully deposited on stainless steel stent platforms as a nanothin microporous coating impregnated with a polymer-free low dose of sirolimus (55 μg). The researchers found no serious adverse cardiac events within a year of follow-up [20].

Mehdizade M. et al. [21] fabricated a magnesium-based biomedical composite by the addition of HAp nanoparticles into a substrate of bioabsorbable WE43 Mg alloy using multi pass friction stir processing. Their results indicated that the Mg-HA-6P bio-composite showed great bioactivity. Grain refined microstructure and presence of HAp particles were responsible for facilitating the formation of bone-like HAp crystals [21]. Akram W. et al. [22] deposited hydroxyapatite on biodegradable pure magnesium. The coatings were analyzed for surface quality, surface roughness, corrosion resistance, and coating adhesion. Both surface roughness and wettability should support cell adhesion. The adhesion to the substrate and wear of the coating are sufficient for the intended biomedical applications. Additionally, the corrosion resistance of magnesium was significantly improved after electrophoretic deposition of hydroxyapatite on it [22].

Erdem U. et al. [23] produced polydopamine coated hydroxyapatite reinforced polyvinyl alcohol film. They prepared composites for potential tissue regeneration. The addition of polydopamine improves interfacial adhesion and provides high mechanical properties, along with preventing the agglomeration of HAp when encountering interstitial fluids. Hydroxyapatite improved the biocompatibility of the composite as it increased the viability values of L929 and MSC cells and antimicrobial properties [23]. Biocompatibility can also be improved by adding carbon materials. Hydroxyapatite composites with carbon allotropes are becoming increasingly important in the field of materials for biomedical applications, especially for strengthening purposes in bone replacement materials. Scientists often use carbon nanotubes or graphene to modify materials. However, composites containing graphite, fullerenes, and nanodiamonds are less popular. There are many ways to obtain hydroxyapatite composites with carbon allotropes, for example, electrochemical and electrophoretic deposition, co-precipitation (with or without ultrasonic treatment), chemical vapor deposition, hydrothermal, spark plasma sintering, and thermal spraying. However, 3D printing, electrospinning, and freeze-drying can be used to produce scaffolds [24]. Han W. et al. [25] presented graphene oxide/hydroxyapatite composite coatings. The electrophoretic deposition method was used to obtain uniform coatings. The composite coatings showed improved adhesion strength and corrosion resistance compared to the HAp coating. Additionally, the obtained material showed antibacterial effects against *S. aureus*. The presence of graphene oxide promoted L929 cell proliferation and mineralization properties. Those studies showed that a graphene oxide content of 5 wt% in the composite was optimal for biomedical applications [25].

There are numerous deposition methods of hydroxyapatite coatings on metallic substrates, e.g., sol–gel, plasma spraying, RF magnetron sputter, pulse laser deposition, electrophoretic deposition, and electrolytic deposition [14,26]. The most popular technique is an electrophoretic deposition [1,22,23,24,25,26,27,28]. The EPD technique has the following advantages: low cost of equipment, coating thickness control, simplicity of the process, capability of coating complex and uniform substrates, and small amount of time needed to obtain the coating [14,29]. This method is based on the dispersion of HAp particles in alcohol, and the electric charge obtained by the particles allows them to deposit on the sample in the form of a layer [1]. Preparation of HAp suspension is commonly based on the HAp particles and a solvent (for example n-butanol, isopropanol). Additionally, it is possible to add a dispersant (triethanolamine, iodine) and other additives, such as PVP (polyvinyl pyrrolidone), PEG (polyethylene glycol), and PEI (polyethylene amine). However, such additives are not desirable since after the drying step, depending on their nature, they may leave residues on the surface of the modified sample [30]. A problem during HAp deposition is the formation of agglomerates. Both the zeta potential and pH of the used suspension are closely related to suspension stability and influence the susceptibility to particle agglomeration. It is also known that the tendency to agglomerate particles is closely related to their size. The literature also states that numerous cracks and agglomerates are noted on coatings produced at higher voltages. Moreover, using a longer deposition time results in a less uniform coating containing more agglomerates and small cracks [31]. Obtaining a stable suspension is one of the possibilities of obtaining agglomerate-free coatings. To make a stable suspension, a dispersant such as triethanolamine (TEA) can be added. X. Xiao et al. [32] obtained the crack-free HAp coating with the addition of TEA as a dispersant. Without adding TEA, the HAp coating was not densely packed and some agglomerated HAp particles were observed on the surface [32]. Polyethylenimine (PEI) can also be used as a dispersing agent [14]. W. Akram et al. [22] used magnetic stirring and ultrasonication, and lowered the pH to 3.5 using acetic acid to obtain a stable suspension [22]. When HAp nanoparticles were suspended in ethanol, agglomerates of HAp nanoparticles were observed on all surfaces [19,33,34]. It is also possible to separate the agglomerates by pre-sedimentation [35].

This work presents research on the surface of nanohydroxyapatite coatings that were prepared on biomedical Ti-13Zr-13Nb alloys by the EPD process using various voltage and time parameters. The parameter values were randomly selected. Sedimentation and removal of sediment from a stable suspension were was used to obtain the agglomerate-free nanohydroxyapatite coatings. No additives, including dispersant agents, were used for this purpose. The properties of the obtained coatings were compared to determine the coating with optimal properties for biomedical applications. The selected coating will be the basis for further modifications in subsequent research.

## 2. Materials and Methods

The electrophoretic deposition method was used to deposit nanohydroxyapatite coatings on Ti-13Zr-13Nb alloy. The titanium samples had the shape of a cylinder with a diameter of 20 mm and a height of 3 mm. The samples were ground with abrasive paper SiC up to grit #2500. Distilled water was used to wash the samples after grinding, and then the surface was dried with compressed air.

The suspension for the deposition was obtained by adding 0.582 g of hydroxyapatite nanopowder, average grain size 20 nm (99% purity, MK Nano, Missisauga, Canada) to 110 mL of ethanol alcohol 99.8% (POCH, Gliwice, Poland) and mixing it in an ultrasonic bath (Sonic-3, Polsonic, Warszawa, Poland) at room temperature for 15 min. In order to minimize the risk of particle agglomeration forming on the surface, primary sedimentation was used. The suspension was left for 72 h for the undissolved fractions to fall to the bottom of ‘beaker I’ and obtain a saturated suspension. After this time, 100 mL of the suspension was taken with a syringe and transferred to another ‘beaker II’. After drying, the amount of sediment was weighed. The final composition of the suspension is 100 mL of ethanol and 0.238 g of nHAp. The suspension was mixed for 15 min in an ultrasonic bath before the electrophoretic deposition process. The coating deposition was performed at room temperature. The deposition parameters are shown in Table 1. The coatings were then dried in a horizontal position at room temperature for 72 h. After that, the samples were placed in a vacuum furnace (PROTHERM PC442, Ankara, Turkey) and heated for 120 min at 800 °C. Such post-deposition heat treatment is intended to increase the density of the coatings and improve the adhesive properties of the nHAp coatings [33,36]. The specimens were cooled in a furnace.

**Figure 1:**
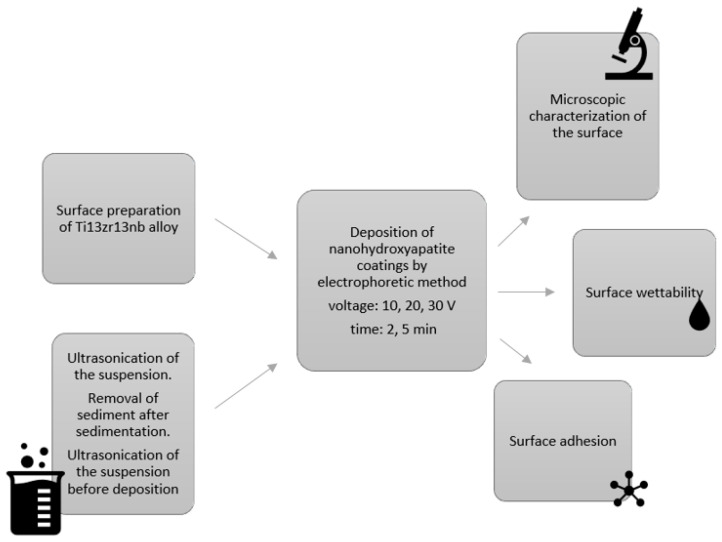
Schematic representation of the sequence of studies described in the article.

The morphology and quality of the coatings were observed using scanning electron microscopy (JEOL JSM-7800F, JEOL Ltd., Tokyo, Japan). To confirm the presence of the coating on the titanium surface, the chemical composition analysis was performed using an energy-dispersive X-ray spectrometer (EDS Edax Inc., Mahwah, NJ, USA). The roughness of the surface was examined using an optical profilometer (Contour GT, 3D Optical Microscope, Bruker, Mannheim, Germany). The thickness of coatings was assessed using an atomic force microscope (Dimension Icon, Bruker, Mannheim, Germany) in tapping mode with RTESPA-300 probes (spring constant 40 N/m, resonance frequency 350 kHz). The nanohydroxyapatite coating on half of the titanium alloy sample was mechanically removed. Its thickness was examined on the border between the coating and the substrate (in the middle of the sample). The sequence of the research is graphically presented in Figure 1.

A drop shape analyzer (Attention Theta Life, Biolin Scientific, Espoo, Finland) was used to assess the wettability of the nanohydroxyapatite coatings. The wettability of the material was evaluated by the falling drop method using water (~3 µL) at room temperature (*n* = 5 for samples). The contact angle (CA) was analyzed for 10 s.

Nanoscratch tests (*n* = 10 for samples) were performed by a nanoindenter (NanoTest Vantage, Micro Materials, Wrexham, United Kingdom). The load increased from 0 mN to 400 mN at the load rate of 2.0 mN/s and at a distance of 500 µm. Both SEM images and changes in frictional force were used to locate the delamination of the coatings. Uncertainties (S) were estimated from standard deviation (S_x_) and experimenter uncertainty (S_e_), where the assumed mistake is 20 mN (S_e_ = 11.5), using (1).

(1) $$S=\sqrt{\frac{1}{3}{S_{e}}^{2}+{S_{x}}^{2}}$$

## 3. Results and Discussion

### 3.1. Microscopic Examinations of Surface

**Figure 2:**
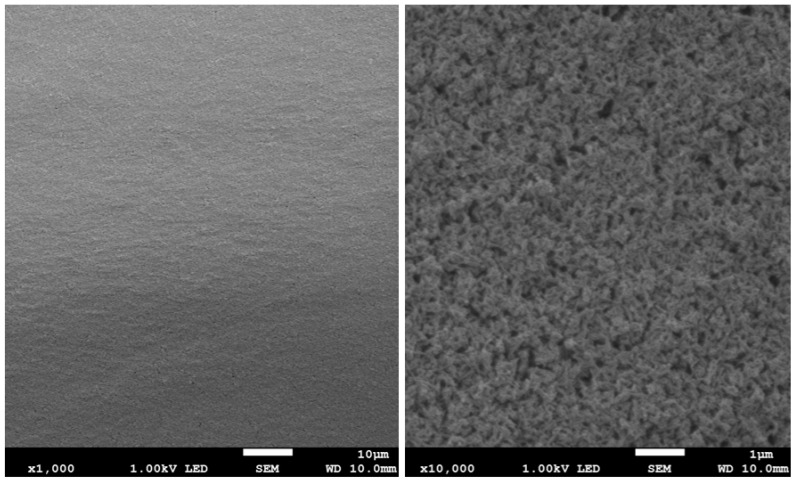
SEM images of nHAp10/2 coating on the Ti-13Zr-13Nb alloy (magnification ×1000 and ×10,000).

**Figure 3:**
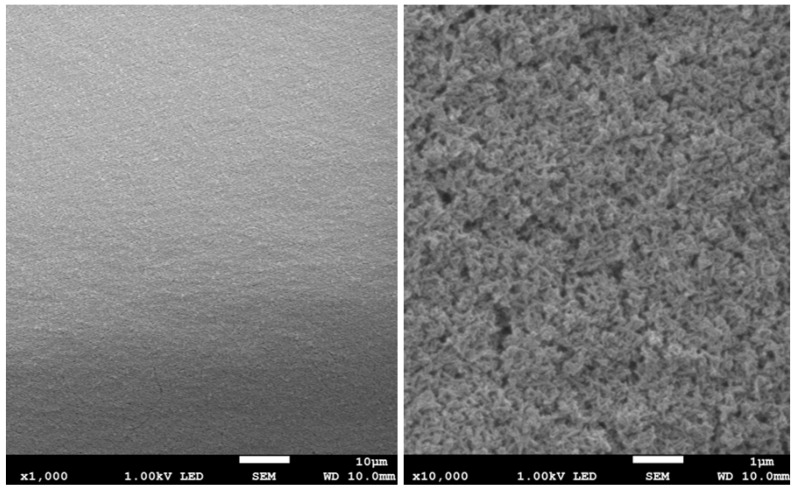
SEM images of nHAp10/5 coating on the Ti-13Zr-13Nb alloy (magnification ×1000 and ×10,000).

**Figure 4:**
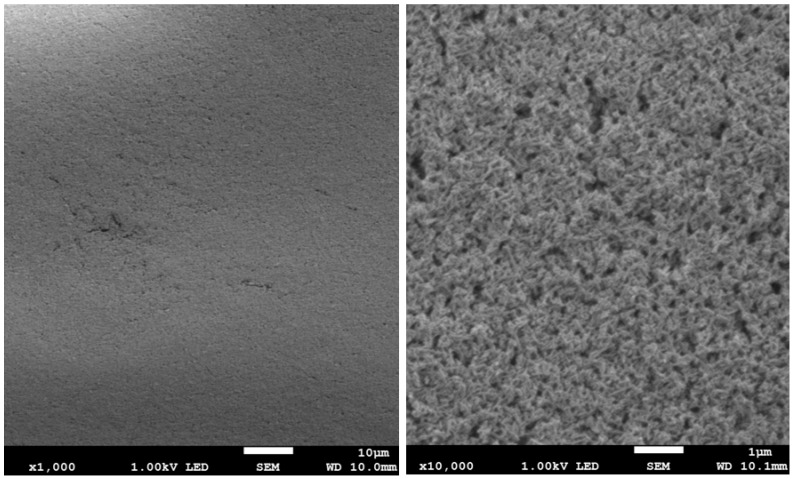
SEM images of nHAp20/2 coating on the Ti-13Zr-13Nb alloy (magnification ×1000 and ×10,000).

**Figure 5:**
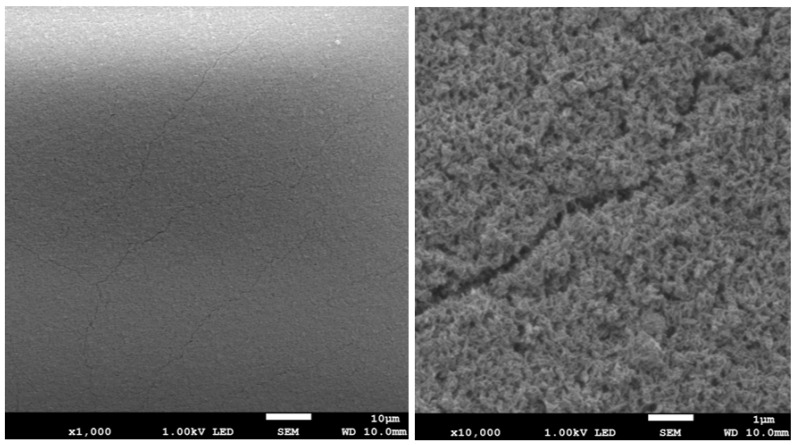
SEM images of nHAp20/5 coating on the Ti-13Zr-13Nb alloy (magnification ×1000 and ×10,000).

**Figure 6:**
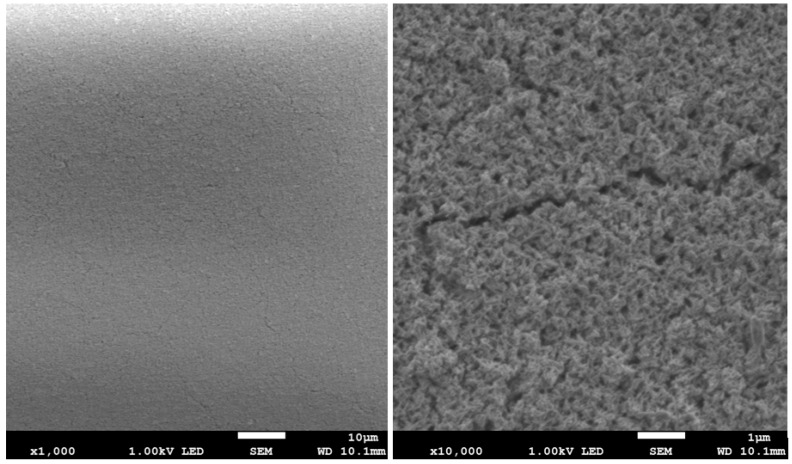
SEM images of nHAp30/2 coating on the Ti-13Zr-13Nb alloy (magnification ×1000 and ×10,000).

**Figure 7:**
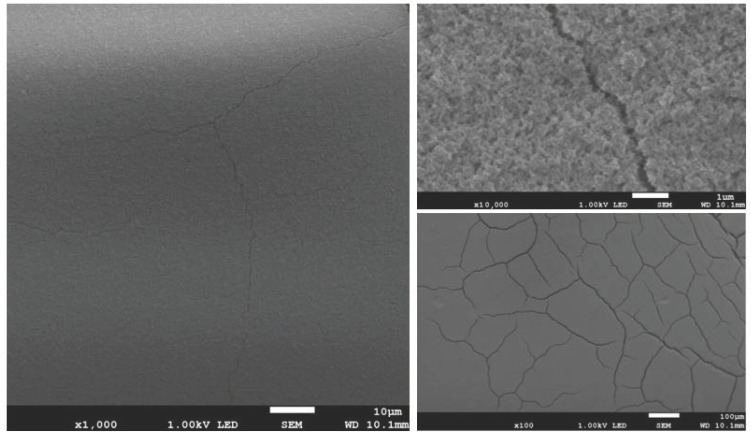
SEM images of nHAp30/5 coating on the Ti-13Zr-13Nb alloy (magnification ×100, ×1000 and ×10,000).

The results of surface imaging using SEM are shown in Figure 2, Figure 3, Figure 4, Figure 5, Figure 6 and Figure 7. No agglomerates of hydroxyapatite nanoparticles were visible on any of the coatings. All coatings are homogeneous. The coatings obtained at a voltage of 10 V were homogeneous and continuous regardless of the deposition time. The increase in voltage caused cracks to appear on the surface.

Hydroxyapatite submicrometer particles exhibit an increased tendency to form agglomerates, a very undesirable occurrence [31]. Scientists most often use dispersing agents to eliminate the possibility of agglomerates forming on the surface of the hydroxyapatite coating [14,32]. After primary sedimentation, no agglomerates formed on the surface. There is information in the literature that a longer deposition time and higher voltage results in a less uniform coating with more agglomerates and cracks. The use of high voltages causes the movement of particles in suspension to be so fast that they will not be able to form a coating of the expected consistency, and the process of their attachment will be chaotic [31]. Regardless of the applied voltage, no hydroxyapatite agglomerates were found on the surface. However, both the increase in voltage and deposition time influence the formation of cracks in the coating. Only when a voltage of 10 V was applied were no cracks observed, regardless of the deposition time used.

**Figure 8:**
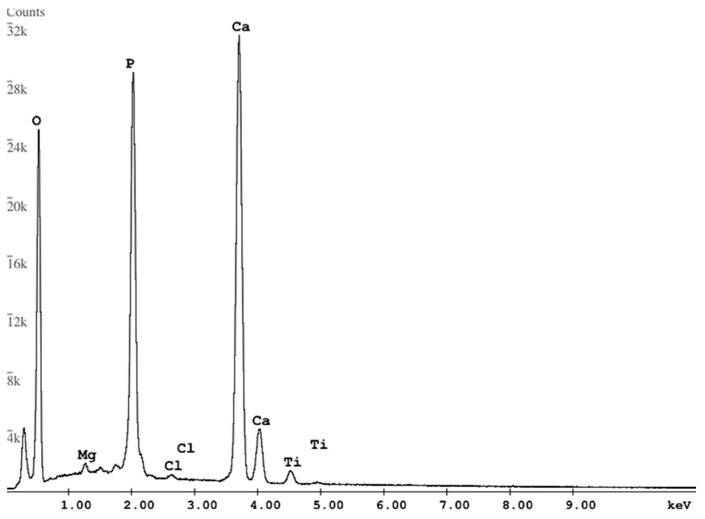
EDS spectrum for nanohydroxyapatite coating.

The qualitative EDS analysis of the sample’s surface (Figure 8) was performed to confirm the presence of a coating on the titanium alloy. EDS revealed the presence of Ti, which is related to the substrate and the elements of which the composite coating was composed (Ca, P, O). A negligible presence of Mg and Cl was also observed. The obtained result is comparable to the literature data [34].

**Figure 9:**
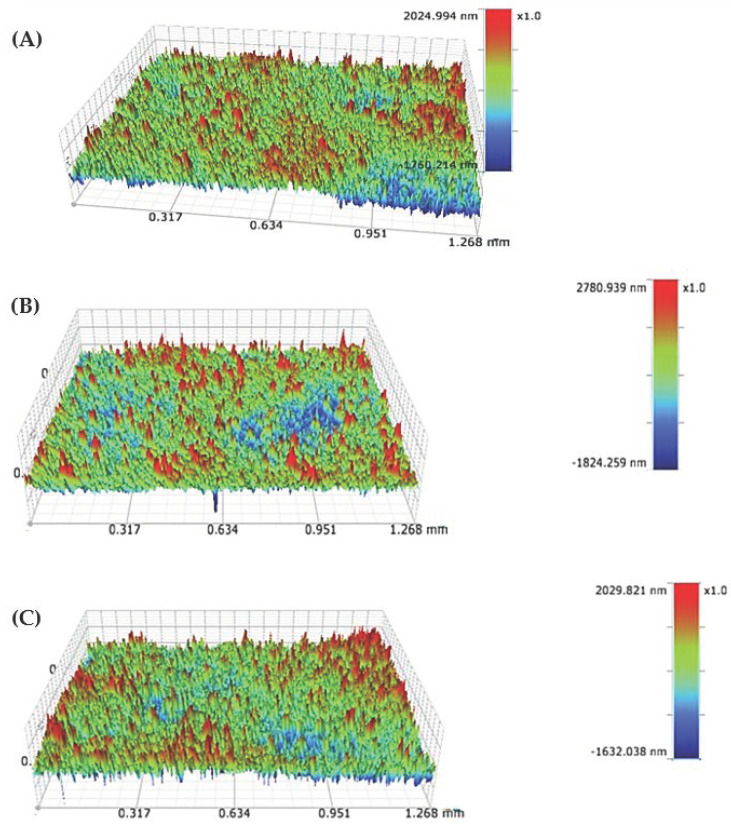
Topographic data from optical profilometry showing surface roughness. (**A**) nHAp10/2, (**B**) nHAp20/2, and (**C**) nHAp30/2.

Surface roughness was examined using an optical profilometer. Results are presented in Table 2 and Figure 9. The assessed parameters are Sa (arithmetical mean height) and Sq (Root mean square height). The coatings have the same chemical composition, which suggests that they should have similar surface roughness, and only surface cracks can significantly affect the parameter values. The obtained surface roughness is lower than is presented in the literature. The Sa parameter for the nHAp coating deposited at 30 V for 2 min was 640 nm [34], with the same parameters obtained in this study Sa is 253 nm, but the higher Sa value may result from the presence of agglomerates on the surface.

The thickness of the sample was measured in its central part. The results of the thickness of nanohydroxyapatite coatings are presented in Table 3. The parameters significantly affect the coating thickness. Both the increase in voltage and the longer deposition time increase the coating thickness. The result for nHAp30/2 (4.8 µm) is comparable to the research presented in the article, where the value for these parameters was 4.7 µm [34].

### 3.2. Wettability Test

**Figure 10:**
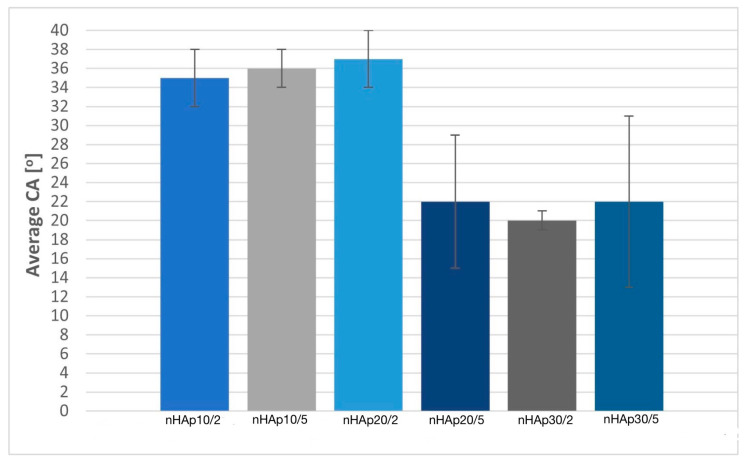
The value of the average contact angle for nanohydroxyapatite samples (*n* = 5).

Wettability tests were performed using the Attention Thete Lite goniometer. The average value of CA (after 10 ± 0.07 s) is presented in Figure 10. All coatings are hydrophilic and the mean contact angle values are in the range of 20–37°. The lowest average CA was obtained in the case of nHAp30/2 (20 ± 1°) and the highest was found in the sample nHAp20/2 (37 ± 3°). Contact angle values are comparable to those reported in the literature. Bartmański et al. deposited HAp (on the same titanium alloy) at a voltage of 30 V for 2 min and the value of the contact angle is about 35.8° [33].

The wettability of biomaterials affects protein adsorption, bacterial and cell adhesion, platelet adhesion, and blood coagulation. The low value of contact angle can correspond to better osseointegration. Numerous pieces of research show that the CA of the coatings should be between 40° and 60° for the best adhesion of cells. This range could vary depending on the cell type. For example, in the case of bone cells in the range of 35–85°, 55° is the ideal value [3,37,38,39]. Samples deposited at 10 V (regardless of time) and 20 V for 2 min are characterized by a contact angle suitable for bone cells.

### 3.3. Nanoscratch Test

**Figure 11:**
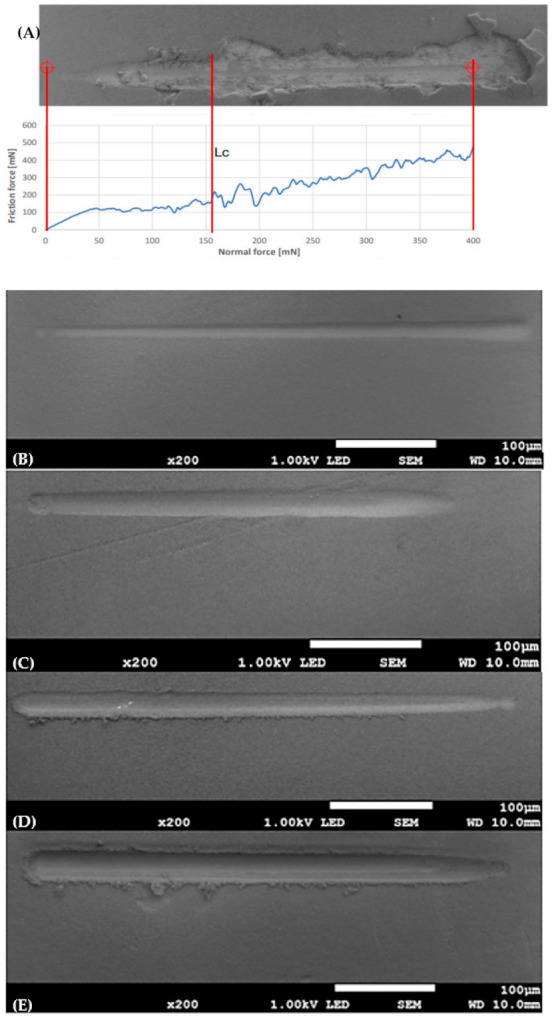
(**A**) Nanoscratch test curves for hydroxyapatite coating (nHAp20/5) with marked delamination force (Lc), and SEM images of (**B**) nHAp10/2, (**C**) nHAp10/5, (**D**) nHAp20/2, and (**E**) nHAp30/2.

The adhesion of the coatings to the metallic substrate was assessed using a nanoscratch test and SEM images. Results are shown in Table 4 and Figure 11. The test was not performed for the nHAp30/5 sample due to the coating crumbling and peeling away. The coatings are characterized by excellent adhesion. Due to the pressure of the indenter, there was no delamination, only smearing of the coating. The scratches visible in SEM images look smoother. Only in the case of nHAp20/5 was there delamination of the coating. The nanoscratch tests presented in the literature brought out the critical load values 36–67 mN for nanoHAp and [37], and other research: 109–124 mN for nanoHAp [40], and 69 mN (nHAp deposition parameters: 50 V, 1 min) [34]. All coatings show better adhesion than those reported in the literature. Adhesion can be influenced by many factors, even the shape of the particles. There are reports that the use of spherical hydroxpapatite particles is associated with better adhesion of the coating to the substrate than in the case of needle or flake shapes. The shape of the particles can also affect the density, uniformity, and hardness of the material. Additionally, a low level of particle dispersion in the suspension leads to the formation of agglomerates, which may impair the adhesion of the coating [31]. The applied suspension stabilization by sedimentation without the addition of dispersant agents could therefore contribute to increasing the adhesion of the coating to the titanium alloy substrate, despite the use of needle-like particles.

## 4. Conclusions

Nanohydroxyapatite coatings were successfully deposited on the Ti-13Zr-13Nb alloy by electrophoretic deposition method. The deposition was carried out by applying voltages of 10, 20, and 30 V for 2 and 5 min. Microscopic examinations, wettability, and nanoscratch tests were performed. The structure was continuous and homogeneous without agglomerations of HAp nanoparticles on the surface. This means that suspension stabilization and sediment removal significantly improved the surface quality. All of the coatings show hydrophilic properties and are characterized by excellent adhesion. Delamination occurred only in the case of nHAp20/5. Due to the lack of agglomerates on the surface, the roughness of the samples is lower than reported in the literature. The range of Sa parameters is 200–280 nm. The most suitable coating for orthopedic implants would be nHAp10/5 because it showed homogeneity, continuity, a contact angle supporting osseointegration (36 ± 2°), great adhesion (in the vast majority of cases, no denomination occurred at the applied force of 400 mN), and a thickness of 4.2 µm without any cracks. The coating will be subject to further modification in future research. The coating will be used as a carrier of drugs and particles to improve antibacterial properties and reduce inflammation in biomedical applications. Based on the results obtained, it is planned to produce composite materials.

## Figures and Tables

**Table 1:** Applied parameters for the deposition of nanohydroxyapatite coatings.

Sample Name	Applied Voltage [V]	Deposition Time [min]
nHAp10/2	10	2
nHAp10/5	10	5
nHAp20/2	20	2
nHAp20/5	20	5
nHAp30/2	30	2
nHAp30/5	30	5

**Table 2:** Results of surface roughness measurements of nanohydroxyapatite coatings.

Sample Name	Sa [nm]	Sq [nm]
nHAp10/2	232	300
nHAp10/5	209	264
nHAp20/2	201	269
nHAp20/5	208	272
nHAp30/2	254	325
nHAp30/5	280	365

**Table 3:** Thickness values of nanoHAp coatings obtained using AFM.

Sample Name	Thickness [µm]	Sample Name	Thickness [µm]	Sample Name	Thickness [µm]
nHAp10/2	1.2	nHAp20/2	3.3	nHAp30/2	4.8
nHAp10/5	4.2	nHAp20/5	5.1	nHAp30/5	7.9

**Table 4:** Nanoscratch test results (*n* = 10).

Sample Name	Critical Load (mN)
nHAp10/2	no delamination
nHAp10/5	no delamination
nHAp20/2	no delamination
nHAp20/5	126 ± 16
nHAp30/2	no delamination
nHAp30/5	not performed

## Data Availability

The original contributions presented in the study are included in the article, further inquiries can be directed to the corresponding authors.
